# Silk Fibroin/Tannin/ZnO Nanocomposite Hydrogel with Hemostatic Activities

**DOI:** 10.3390/gels8100650

**Published:** 2022-10-12

**Authors:** Chul Min Yang, Jeehee Lee, Su Yeon Lee, Haeshin Lee, Kiramage Chathuranga, Jongsoo Lee, Wonho Park

**Affiliations:** 1 Department of Organic Materials Engineering, Chungnam National University, Daejeon 34134, Korea; 2 Department of Chemistry, Korea Advanced Institute of Science and Technology (KAIST), Daejeon 34141, Korea; 3 Department of Veterinary Microbiology, College of Veterinary Medicine, Chungnam National University, Daejeon 34134, Korea

**Keywords:** tannic acid, silk fibroin, zinc oxide nanoparticle, composite hydrogel, hemostatic

## Abstract

The inevitable bleeding and infections caused by disasters and accidents are the main causes of death owing to extrinsic trauma. Hemostatic agents are often used to quickly suppress bleeding and infection, and they can solve this problem in a short time. Silk fibroin (SF) has poor processibility in water, owing to incomplete solubility therein. In this study, aiming to overcome this disadvantage, a modified silk fibroin (SF-BGE), easily soluble in water, was prepared by introducing butyl glycidyl ether (BGE) into its side chain. Subsequently, a small amount of tannic acid (TA) was introduced to prepare an SF-BGE /TA solution, and ZnO nanoparticles (NPs) were added to the solution to form the coordination bonds between the ZnO and TA, leading to an SF-based nanocomposite hydrogel. A structural characterization of the SF-BGE, SF-BGE/TA, SF-BGE/TA/ZnO, and the coordination bonds between ZnO/TA was observed by attenuated total reflectance-Fourier transform infrared spectroscopy (ATR-FTIR), and the phase change was observed by rheological measurements. The pore formation of the SF-BGE/TA/ZnO hydrogel and dispersibility of ZnO were verified through energy-dispersive X-ray spectroscopy (EDS) and scanning electron microscopy (SEM). The cytocompatible and hemostatic performances of the SF-BGE/TA/ZnO NPs composite hydrogels were evaluated, and the hydrogels showed superior hemostatic and cytocompatible activities. Therefore, the SF-based nanocomposite hydrogel is considered as a promising material for hemostasis.

## 1. Introduction

The unavoidable hemorrhage and infections caused by disasters and accidents are the main causes of death owing to extrinsic trauma [1,2]. In addition, severe wounds are susceptible to infections, which can disrupt the natural healing process and lead to life-threatening sepsis. However, the development of a hemostatic agent for effectively suppressing bleeding and infection remains a difficult task [3]. In general, the hemostatic agents of traditional wound dressings (in the form of bandages such as gauze dressings) are not suitable for application to irregularly shaped wounds with bleeding, owing to their low hemostatic effects. Therefore, the development of a multifunctional hemostatic agent with facile and effective hemostatic activities properties is urgently required [4,5]. 

*Bombyx mori* silk is a natural fiber consisting of two proteins (fibroin and sericin), and it has excellent biocompatibility, biodegradability, and mechanical performance [6,7,8]. In addition, silk fibroin (SF) can improve the cell adhesion properties of biomaterials. In this context, SF is being studied as a hemostatic material because the blood clotting activity increases and bleeding time decreases when SF is added to a hemostatic agent [9]. However, an SF solution is generally disadvantageous, owing to difficulties in water solubility and storage [10,11,12]. Tannic acid (TA) is a type of natural polyphenol widely present in various plants. It is easily extracted, and it has been approved by the Food and Drug Administration (FDA) [13]. It has useful properties (e.g., antioxidant, hemostatic, and antibacterial properties), owing to its many phenolic hydroxyl groups [14,15]. In addition, TA can complex or crosslink polymers through various intermolecular interactions, such as hydrogen bond, coordination bond, ionic bond, and hydrophobic interaction. Therefore, TA is an ideal gelator for hydrogel formation [16,17]. For example, TA is very useful as a crosslinking agent for providing polymer bioadhesiion [18]. ZnO is also a bioactive agent approved by the FDA, and it has antibacterial properties against a wide range of pathogens including bacterial strains and fungi. Moreover, ZnO nanoparticles (ZnO NPs) show an antibacterial activity distinct from that of large particles [19,20]. ZnO NPs are also used as drug carriers, fillers in cosmetics, and in medical devices, owing to their non-toxicity, biostability and biocompatibility. Although some papers have reported the harmful effects of ZnO NPs, low concentrations of ZnO NPs are not toxic to human cells [21,22]. Zinc has been reported to have excellent wound-healing and bleeding-inhibition performances when applied as a hemostatic agent [23]. 

**Figure 1:**
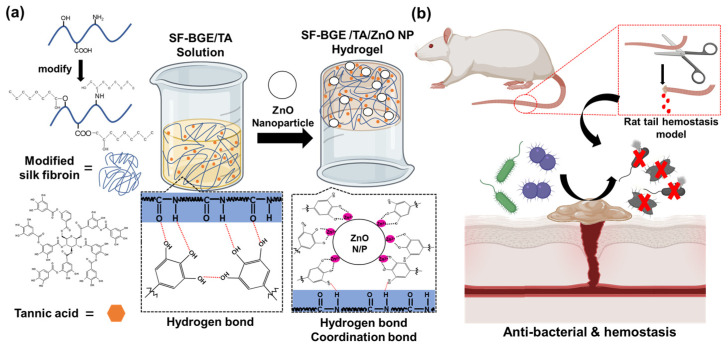
(**a**) Schematic of the modified SF (SF-BGE), tannic acid (TA), ZnO nanoparticles (NPs), SF-BGE/TA solution, and SF-BGE/TA/ZnO hydrogel hemostasis. (**b**) Schematic of the hemostasis trials in the rat tail amputation model and antibacterial and hemostatic process of SF-BGE/TA/ZnO NPs.

In this study, a composite hydrogel consisting of modified SF (SF-BGE), TA, and ZnO NPs was prepared by consecutive mixing. Figure 1 shows the formation process and functionalities of SF-BGE/TA/ZnO composite hydrogel. Firstly, SF was reacted with butyl glycidyl ether (BGE) to obtain a water-soluble SF-BGE derivative. The SF-BGE/TA solution was prepared by varying the mixing ratio of the SF-BGE and TA. Subsequently, a sol–gel transition induced coordination bondings between the TA and ZnO by adding ZnO NPs to the SF-BGE/TA solution. A rat tail amputation model was employed to investigate the hemostatic effect of the SF-BGE/TA/ZnO composite hydrogel.

## 2. Results and Discussion 

### 2.1. Characterization and Rheology of SF-BGE, SF-BGE/TA, and SF-BGE/TA/ZnO NP Hydrogel

**Figure 2:**
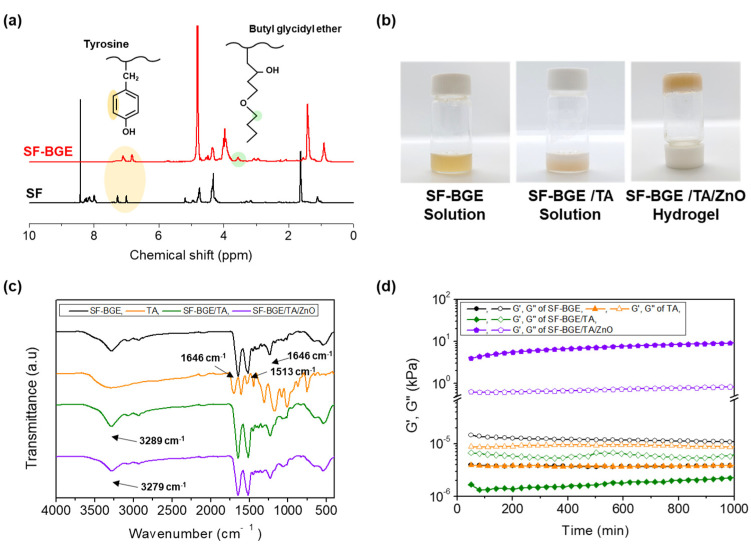
(**a**) ^1^H NMR spectra of pristine SF and SF-BGE. (**b**) Photographs demonstrating of SF-BGE and SF-BGE/TA solutions and SF-BGE/TA/ZnO hydrogel. (**c**) ATR-FTIR spectra of SF-BGE, TA, SF-BGE/TA, and SF-BGE/TA/ZnO hydrogel. (**d**) G′ and G″ of SF-BGE, TA, SF-BGE/TA, and SF-BGE/TA/ZnO hydrogel.

The DS (%) of SF-BGE was determined from ^1^H-NMR spectrum (Figure 2a). The resonance peak of the aromatic ring of the tyrosine residue in the SF was observed at δ = 6.7–7.3 ppm, and the resonance peak of the alkyl group next to ether group of BGE was observed at δ = 3.4–3.7 ppm. The DS (%) of the SF-BGE was determined as ~31.6% [24]. The structural characterizations of the SF-BGE, TA, and SF-BGE/TA solutions and SF-BGE/TA/ZnO hydrogel were conducted using ATR-FTIR spectroscopy (Figure 2c). The SF-BGE, SF-BGE/TA solution, and SF-BGE/TA/ZnO hydrogel exhibited three absorption peaks: amide I at 1646 cm^−1^, amide II at 1513 cm^−1^, and amide III at 1233 cm^−1^. The absorption of the SF-BGE at 3200 cm^−1^ corresponds to N-H stretching [25,26]. The broad peak of TA at 3285 cm^−1^ is attributed to the stretching vibration of -OH. In addition, the absorption peaks at 1692 and 1176 cm^−1^ correspond to the conjugated C=O and C–O stretching vibrations, respectively, and the absorption peaks at 1605, 1529, and 1442 cm^−1^ are related to the aromatic ring of TA [27]. In the SF-BGE/TA solution, the absorption peak at 3289 cm^−1^ corresponds to the stretching vibration peak of the –OH groups of TA, and the absorption peak of –OH shifts to 3279 cm^−1^ in the SF/TA/ZnO hydrogel. This shift in the absorption peak is owing to the formation of coordination bonds between the ZnO and TA [28].

The rheological properties of the SF-BGE, TA, and SF-BGE/TA solutions and SF-BGE/TA/ZnO hydrogel were examined using a rheometer (Figure 2d). The effect of TA on the SF-BGE solution and the effect of ZnO NPs on the SF-BGE/TA solution were interpreted in detail according to the TA and ZnO Nps contents via rheometry [29]. In the SF-BGE, TA, and SF-BGE/TA solutions, the storage modulus (G′) was lower than the loss modulus (G″), indicating that they were in solution state. In particular, the SF-BGE/TA sample maintained the solution phase without the formation of a coacervate, since a small amount of TA was added to form weak hydrogen bonds. However, the G′ was higher than the G″ in the SF-BGE/TA/ZnO hydrogel, indicating that a composite hydrogel was formed. By adding the ZnO NPs, the SF-BGE/TA solution with weak hydrogen bonds changed to a hydrogel by forming a network structure as a whole via coordination bondings between the ZnO and TA. Photographs of the SF-BGE and SF-BGE/TA solutions and SF-BGE/TA/ZnO hydrogel are presented in Figure 2b

### 2.2. Morphology of SF-BGE and SF-BGE/TA Solutions and SF-BGE/TA/ZnO NP Hydrogel 

**Figure 3:**
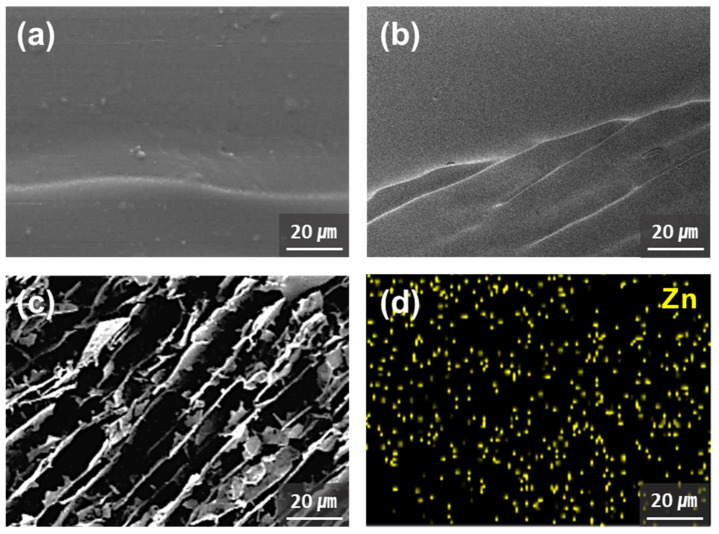
SEM images of (**a**) SF-BGE, (**b**) SF-BGE/TA, and (**c**) SF-BGE/TA/ZnO hydrogel. (**d**) EDS mapping image of the SF-BGE/TA/ZnO hydrogel.

The morphologies of the SF-BGE and SF-BGE/TA solutions and SF-BGE/TA/ZnO hydrogel were observed after freeze-drying, and SEM images of the cross-sections are shown in Figure 3a–c. The SF-BGE/TA/ZnO composite hydrogel contained many pores, whereas the SF-BGE and SF-BGE/TA samples had almost no pores. These pores were generated by the evaporation of water from the hydrogel during freeze-drying. In contrast, the ZnO NPs exhibited low dispersibility in the polymer matrices, owing to their high specific surface energy and polar surface [30]. EDS mapping was performed to analyze the dispersion and aggregation of ZnO NPs in the SF-BGE/TA/ZnO hydrogel (Figure 3d). The yellow signal in the EDS mapping image indicates Zn, which was uniformly distributed without severe agglomeration. This indicates that the ZnO formed coordination bonds with the TA molecules, leading to hydrogel formation.

### 2.3. Cytocompatibility of SF-BGE, TA, and SF-BGE/TA Solutions and SF-BGE/TA/ZnO NP Hydrogel

**Figure 4:**
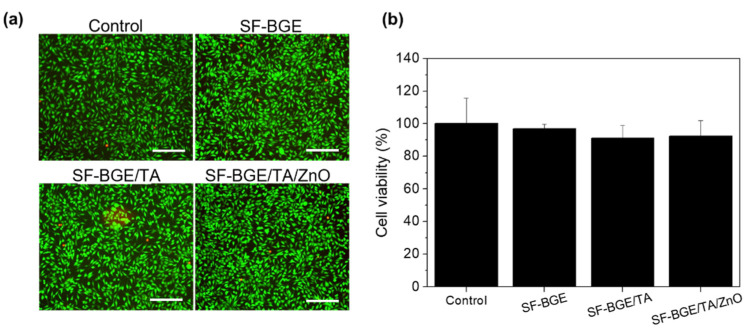
Indirect cytotoxicity test of SF-BGE, SF-BGE/TA, and SF-BGE/TA/ZnO samples on L929 fibroblast with (**a**) LIVE/DEAD staining (green: live, red: dead) and (**b**) cell viability (%) quantified based on the cell counting kit-8 (CCK-8) assay using extracts of SF-BGE and SF-BGE/TA solutions and SF-BGE/TA/ZnO hydrogel for 24 h. As a control, Dulbecco’s phosphate-buffered saline (DPBS) was used (inset scale bar is 200 µm). The data are expressed as the mean ± standard deviation (n = 3). All samples are not significantly different (per one-way analysis of variance (ANOVA) and Tukey’s post hoc test).

To examine the cytocompatibility of SF-BGE and SF-BGE/TA solutions and SF-BGE/TA/ZnO hydrogel, indirect cell viability tests were performed with a LIVE/DEAD assay for imaging and CCK-8 for quantifying after incubation for 24 h. The number of dead (red) fibroblasts (L929 cells) did not significantly decrease, and the cells did not exhibit round or dying morphologies (Figure 4a). The cell viability of all samples was above 90%: SF-BGE (97 ± 2%), SF-BGE/TA (90 ± 7%), and SF-BGE/TA/ZnO 10 (92 ± 9%) (Figure 4b). The SF-BGE/TA/ZnO hydrogel was determined to be non-toxic (grade 0) based on the ISO 10993-5 standard. In general, ZnO induces apoptosis via Zn^2+^, and it is known to be cytotoxic [31]. However, it was expected that the SF-BGE/TA/ZnO hydrogel would not show cytotoxicity because the eluted Zn^2+^ formed coordination bonds with the TA. Based on these results, the SF-BGE/TA/ZnO hydrogel with a low cytotoxicity is suitable for hydrogel-type hemostatic applications.

### 2.4. In Vivo Hemostatic Capability of SF-BGE and SF-BGE/TA Solutions and SF-BGE/TA/ZnO Hydrogel with Rat Tail Amputation Model

**Figure 5:**
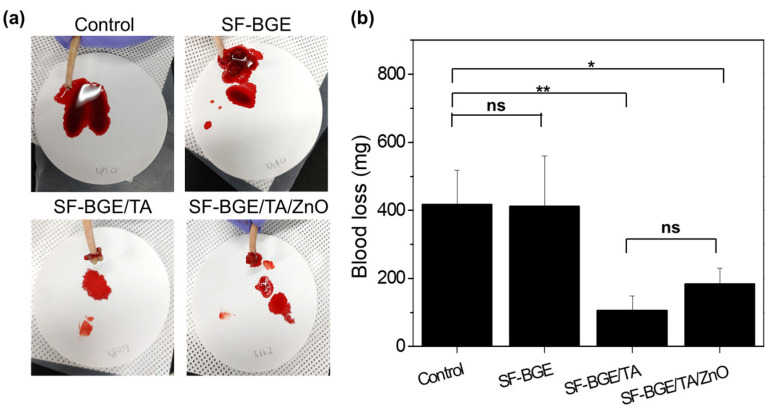
(**a**) Photographs of blood loss after tail amputation and administration of SF-BGE, SF-BGE/TA and SF-BGE/TA/ZnO hydrogel. (**b**) Blood loss (mg) from injury rat tail of control, SF-BGE, SF-BGE/TA, and SF-BGE/TA/ZnO hydrogel. The results are reported as the mean ± standard deviation (n = 3). Statistical significance was analyzed by one-way ANOVA and Tukey’s post hoc test (* *p* < 0.05, ** *p* < 0.01).

The hemostatic capability of the SF-BGE/TA/ZnO hydrogel was evaluated from a rat tail amputation model rather than a liver hemostasis model, so as to mimic the administration route of hemostatic agents from the outer skin. The SF-BGE/TA solution and SF-BGE/TA/ZnO hydrogel attached to the surface of the tail incision effectively blocked the bleeding (Figure 5a). However, the SF-BGE did not adhere to the surface of the tail incision, resulting in no effect on hemostasis. In contrast, the SF-BGE/TA and SF-BGE/TA/ZnO exhibited significant reductions in the bleeding amount, from 416.9 ± 115.2 mg (control) and 412.5 ± 130.9 mg (SF-BGE) to 105.5 ± 57.1 mg and 183.4 ± 50.1 mg, respectively (Figure 5b). This hemostasis might be attributed to the tissue adhesion and hemostatic capabilities of the TA [32,33]. In view of the reduction in blood loss, the SF-BGE/TA/ZnO hydrogel showed an excellent hemostatic performance with the TA. Moreover, it can be expected that the SF-BGE/TA/ZnO hydrogel can be even applied in the wound healing, owing to its hemostatic and antibacterial activities.

## 3. Conclusions

In this study, an SF-based hemostatic hydrogel with excellent cytocompatibility was proposed. TA and ZnO NPs were added to the SF solution to provide hemostatic properties, and to form a nanocomposite hydrogel. Firstly, the SF was chemically modified with BGE to improve its water solubility. Thereafter, the SF-BGE/TA solution was prepared by adding TA to induce hydrogen bonds between the SF-BGE and TA, and ZnO NPs were added to prepare the nanocomposite hydrogel via coordination bonds between the ZnO and TA. The structural and morphological characterizations of SF-BGE, SF-BGE/TA, SF-BGE/TA/ZnO and the coordination bonds between TA/ZnO were verified by ATR-FTIR and SEM-EDS. In addition, the SF-BGE/TA/ZnO hydrogel exhibited excellent cell viability over 90%, and it was judged as non-toxic according to the ISO 10993-5 standard. Moreover, the SF-BGE/TA/ZnO hydrogel showed excellent tissue adhesion and hemostatic properties in a rat tail amputation model, although its hemostatic activity was slightly lower than that of SF-BGE/TA solution owing to coordination bonds between TA and ZnO. If both TA and ZnO NPs are more activated by tuning their mixing ratio, the SF-BGE/TA/ZnO hydrogel with low cytotoxicity is considered as suitable for application as a hemostatic hydrogel material.

## 4. Materials and Methods

### 4.1. Materials

The SF (*Bombyx mori*) was supplied by Da Sung Silk Co., Ltd. (Jinju-si, Korea). The TA (M_n_ = 1701), ZnO NP (size < 100 nm), and BGE (purity, 95%) were obtained from Sigma-Aldrich Co. (St. Louis, MO, USA). Ethyl alcohol (EtOH; purity, 99.5%), calcium chloride dihydrate (CaCl_2_; purity; 71.0–77.5%) were supplied by Samchun Chemical Co., Ltd. (Pyeongtaek-si, Korea). All of the chemicals were used as received.

### 4.2. Preparation of Water-Soluble Silk Fibroin (SF)-Butyl Glycidyl Ether (BGE) Derivative

The synthesis of the SF derivatives was conducted as reported in previous studies [24]. Briefly, the degummed SF was dissolved in CaCl_2_/EtOH/H_2_O (1/2/8, molar ratio) at 70 °C for 1 h. The 20 mL of BGE was added dropwise into the SF solution at a rate of 0.5 mL/min to avoid aggregation, and the resultant mixture was reacted at 70 °C for 3 h. After the complete reaction, the solution containing the SF-BGE product was dialyzed in distilled water to remove unreacted BGE using a cellulose dialysis membrane (MW cut-off, 14,000 Da) for 5 days. Thereafter, the impurities were removed by centrifugation at 3000 rpm for 10 min, and the resultant SF-BGE solution was freeze-dried at −80 °C for 7 days. The freeze-dried SF-BGE derivative was kept at 25 °C prior to use. The product was notated as “SF-BGE”. The modification of SF was verified using proton nuclear magnetic resonance (^1^H NMR) spectroscopy. For the NMR analysis, the pristine SF and SF-BGE were dissolved in deuterated formic acid (DCOOD) (Sigma-Aldrich Co., St. Louis, MO, USA) and D_2_O (Sigma-Aldrich Co., St. Louis, MO, USA), respectively. The degree of substitution (DS, %) of the SF-BGE was determined using the hydrogen peak of the aromatic ring in the tyrosine residue of the SF (δ = 6.7–7.3 ppm) and hydrogen peak bonded to the carbon next to the ether group in the BGE (δ = 3.4–3.7 ppm), as follows:

(1) $$DS\;\left(\%\right)=\frac{P_{BGE}}{\left(P_{Tyr/4}\right)\times \frac{100}{11.11}}\times \frac{100}{mole\%\;of\;reactive\;groups}\times 100$$

### 4.3. Preparation of SF-BGE/TA/ZnO NP Composite Hydrogel

First, the SF-BGE (20 wt%) and TA (1.3 wt%) were dissolved in distilled water. The TA solution was added dropwise to the SF-BGE solution and stirred at room temperature for 2 h to obtain the SF-BGE/TA solution. Subsequently, the ZnO NPs were added to the SF-BGE/TA solution at a TA:ZnO molar ratio of 2:10 to form coordination bonds between the ZnO and TA. Subsequently, the SF-BGE/TA/ZnO composite hydrogel was obtained after stabilization for 6 h.

### 4.4. Structural Analyses

The structural characterizations of the SF-BGE, TA, and SF-BGE/TA solutions and the SF-BGE/TA/ZnO hydrogel were conducted by attenuated total reflectance-Fourier transform infrared spectroscopy (ATR-FTIR; ALPHA-P, Bruker, Billerica, MA, USA). The spectrum was obtained in the spectral range of 4000–400 cm^−1^ with a resolution of 4 cm^−1^. Each sample was freeze-dried and measured in the solid state.

### 4.5. Rheological Measurement

To analyze the rheological properties of the SF-BGE, TA, and SF-BGE/TA solutions and the SF-BGE/TA/ZnO hydrogel, a rotational HAAKE MARS-40 rheometer (Thermo Fisher Scientific Inc., Kalsruhe, Germany) with Peltier plate geometry (35 mm diameter, 1.2 mm gap) was used. The storage modulus (G′) and loss modulus (G″) represented the elastic and viscous behaviors of the polymer, respectively. A time-sweep oscillatory test was conducted in the linear viscoelastic region of the G′ and G″ at a stain of 0.1% for 900 s at 20 °C.

### 4.6. Scanning Electron Microscopy and Energy Dispersive X-ray Spectroscopy

The surface morphologies of the SF-BGE, SF-BGE/TA, and SF-BGE/TA/ZnO samples were observed by field-emission scanning electron microscopy (FE-SEM; Carl Zeiss, Merlin compact, Oberkochen, Gemany). Each specimen was cut after lyophilization using freeze-dryer (FD 8508, IlshinBioBase, Daejeon, Korea), and its surface was coated with platinum for the SEM observation. The distribution of the ZnO NPs in the composite hydrogel was observed through energy-dispersive X-ray spectroscopy (EDS).

### 4.7. Cell Viability

The L929 cells (1 × 10^4^ cells/well) were prepared with Dulbecco’s Modified Eagle Medium (pH 7.4, Gibco; Thermo Fisher Scientific, Inc., Waltham, MA, USA) as complemented with 10 vol% fetal bovine serum and 1 vol% penicillin and streptomycin in 96 well plates. To conduct an indirect cytotoxicity test, the SF-BGE/TA/ZnO sample was placed in Dulbecco’s PBS (DPBS, pH 7.4, Gibco; Thermo Fisher Scientific, Inc., Waltham, MA, USA) (10 mg/mL) at 37 °C. Both the cells and extracts were incubated in the 37 °C and 5% CO_2_ environment for 24 h. Sample extracts (10 μL) were added to each well. After exposure for 24 h, cytotoxicity was measured by qualification and quantification experiments. For imaging, the culture media of the cells were removed, and the cells were stained using a LIVE/DEAD^®^ Viability/Cytotoxicity Kit (Invitrogen, Carlsbad, CA, USA) containing calcein AM (2 μM) and ethidium homodimer (4 μM) prepared in DPBS for 10 min. Fluorescence images were obtained using a fluorescence microscopy (Eclipse Ti; Nikon, Tokyo, Japan). For quantification, the cell counting kit-8 (CCK8) was utilized. After exposure for 24 h, 10 μL of CCK8 was applied to each well and incubated in the 37 °C and 5% CO_2_ environment for 1 h. The absorbance at 450 nm was captured using the Varioskan Flash reader (Thermo Fisher Scientific Inc., Waltham, MA, USA). All of the experiments were conducted in triplicate, and cell viability (in the context of indirect cytotoxicity) was determined according to the International Organization for Standardization (ISO) 10993-5 standard.

### 4.8. Rat-Tail Amputation for Testing Hemostatic Capability In Vivo

The animal experiments were performed under the approval of the Animal Care Committee of KAIST (KA2021-078). To verify the hemostatic capability of the SF-BGE/TA/ZnO sample, 8-week-old male Sprague Dawley rats (Orient Bio, Seongnam-si, Korea, 275–325 g, n = 3 to 4) were anesthetized with Zoletil and Rompun by an intramuscular route. The tail amputation was induced by cutting the tail at a point 2 cm from the tip. After bleeding for 10 sec, the SF-BGE, SF-BGE/TA, or SF-BGE/TA/ZnO samples (200 mg) were applied on the incisions of the tails using a spatula. The amount of blood loss (mg) was measured by weighing the blood absorbed on pre-weighed filter paper for another 2 min. As a control, in one sample, nothing was applied to the tail incision. To prevent the evaporation of blood, the mass of blood was weighed within a few seconds. After the experiment, the animals were sacrificed in a CO_2_ chamber. The SF-BGE and SF-BGE/TA solutions were stored at 4 °C for 5–7 days to prepare a high-viscosity liquid or gel to facilitate the measurement of the hemostatic capacity.

### 4.9. Statistical Analysis

All experiments were repeated at least three times unless otherwise described. The results were statically analyzed by one-way analysis of variance (ANOVA) followed by Tukey’s honest significant difference test (*p* < 0.05).

## Data Availability

The data presented in this study are available in this article.
